# The association between problematic internet use and neck pain among Japanese schoolteachers

**DOI:** 10.1002/1348-9585.12298

**Published:** 2021-12-09

**Authors:** Rina Tanabe, Takashi Hisamatsu, Mari Fukuda, Hideki Tsumura, Rina Tsuchie, Masako Suzuki, Nagisa Sugaya, Koshi Nakamura, Kenzo Takahashi, Hideyuki Kanda

**Affiliations:** ^1^ Department of Public Health Okayama University Graduate School of Medicine, Dentistry, and Pharmaceutical Sciences Okayama Japan; ^2^ Department of Psychology Faculty of Integrated Arts and Sciences Tokushima University Tokushima Japan; ^3^ Department of Nursing Faculty of Medicine Shimane University Shimane Japan; ^4^ Department of psychology Faculty of Education and Humanities Jumonji University Saitama Japan; ^5^ Unit of Public Health and Preventive Medicine Faculty of Medicine Yokohama City University Kanagawa Japan; ^6^ Department of Public Health and Hygiene Graduate School of Medicine University of the Ryukyus Okinawa Japan; ^7^ Teikyo University Graduate School of Public Health Tokyo Japan

**Keywords:** internet addiction, musculoskeletal pain, neck pain, problematic internet use, teachers

## Abstract

**Objectives:**

Problematic internet use (PIU) has been suggested in relation to psychological symptoms among schoolteachers, but the relationship with physical symptoms remains unclear. We examined whether PIU or longer Internet usage time is associated with neck pain in schoolteachers.

**Methods:**

We conducted a cross‐sectional study among 2582 teachers aged 20 years or older (35.6% women) in Shimane and Tottori, Japan in 2018. Neck pain was defined as ≥5 points on the Neck Disability Index. The Compulsive Internet Use Scale (CIUS) was used to assess PIU. Internet usage time on weekdays and weekends was divided into five groups: 0, 1–29, 30–59, 60–119, and ≥120 min/day. Logistic regression analysis was conducted to examine the association of the CIUS score and Internet usage time on weekdays or weekends with neck pain, adjusting for sex, age, position at school, insomnia, and psychological distress.

**Results:**

We observed 800 (31.0%) teachers with neck pain. The median (interquartile range) of their CIUS scores was 7 (2, 14). A higher CIUS score was independently associated with a higher prevalence of neck pain (odds ratio of 4th vs. 1st quartiles, 1.41; 95% confidence interval, 1.06–1.87; trend *P* = .006). Compared with non‐Internet users, Internet users on weekdays had almost double the odds of neck pain although the difference did not reach the customary level for designating statistical significance.

**Conclusions:**

In conclusion, teachers with higher scores in CIUS were associated with a higher prevalence of neck pain in Japan, suggesting adults with PIU being at risk of physical disorders.

## 1 INTRODUCTION

The Internet is used by about 53% of the global population, or 4.1 billion people, based on the 2019 ICT statistics. The number of Internet users grew on average by 10% every year between 2005 and 2019.^1^, ^2^ Increasing popularity and frequency of Internet use has led to the emergence of problematic internet use (PIU). Today PIU is understood to be uncontrollable and damage with addictive use of the Internet.^3^, ^4^ Previous studies have reported that the prevalence of PIU is up to approximately 25% for adolescents and 23% for adults, worldwide.^5^ Due to the markedly increased use of the Internet over the past 15 years, PIU has attracted the attention of researchers and clinicians in the field.

Previous studies in different countries have shown that teaching is one of the most stressful occupation,^6^, ^7^, ^8^ and its major cause is long working hours.^9^ According to the OECD survey announced in 2018, average working hours per week of junior high school teachers was 56.0 in Japan, which is the longest across 48 countries and regions (its average of all countries and regions, 38.3 h per week).^10^ Longer working hours were also associated with mental health among teachers.^9^ Burnout syndrome is one related symptom of such a mental state and has attracted much research attention for decades,^11^ and this syndrome has a positive correlation with PIU in such populations.^3^

Globally, neck pain was the primary cause of disability and years lived with disabilities (YLDs) in 2015.^12^ Among 25 to 64‐year‐old people, it was also the leading cause of global age‐specific YLDs from 2005 to 2015. In addition, physical disorders including musculoskeletal pain have been reported in relation to PIU,^13^, ^14^ and adolescents with longer Internet use have had more musculoskeletal symptoms.^15^ Despite the recently increasing trend in burden of PIU or Internet use in schoolteachers,^16^ to our knowledge, few studies have evaluated whether PIU or excessive Internet usage are associated with musculoskeletal pain in such populations.

Therefore, the purpose of the present study was to examine whether PIU or longer duration of internet use is associated with neck pain, which is mostly prevalent in self‐reported musculoskeletal symptoms^17^, ^18^ in schoolteachers aged 20 years or older.

## 2 METHODS

### 2.1 Study participants

This study was a prefecture‐wide cross‐sectional design using an anonymous self‐administered survey.^3^, ^14^, ^16^ The target population was 9990 school personnel (i.e., school staff including administrators, teachers, clerks) in all 241 junior and senior high schools in Shimane and Tottori Prefectures, rural areas in Japan. In August 2018, we sent the study information and questionnaires to all junior and senior high schools in Shimane and Tottori Prefectures and asked all personnel to participate in the study in the schools whose principals consented to participation. They were informed that the survey was voluntary, and that anonymity and confidentiality were ensured. Each staff member sealed their completed questionnaires in the envelope, and the administrators collected and returned them to us unopened in September 2018. A completed questionnaire was considered as consent to the study.

Among the 241 schools, 119 schools consented to participate in the survey (response rate: 49.4%). Among 9990 school staff members in these schools, 3314 responded to the survey (response rate: 33.2%). Among them we excluded 505 clerical or technical workers and 227 with missing data. Therefore, data from 2582 eligible teachers (age range, 20 years or older; 919 [35.6%] women) were included in the analysis (eligible response rate: 77.9%). This survey was approved by the Institutional Review Board of Shimane University (no. 3274, Approved on July 24, 2018) and Okayama University (no. 2020‐027, Approved on January 31, 2020).

### 2.2 Assessment of neck pain

Neck pain was measured by the Neck Disability Index (NDI), which is the most widely used and most strongly validated instrument to assess self‐rated disability in patients with neck pain.^19^ The NDI is a 10‐item test, scored from 0 to 5 (with a possible sum of scores from 0 to 50). Higher scores represent a more severe state of neck pain. The total score classifies neck pain as follows: No disability (0–4 points), Mild (5–14 points), Moderate (15–24 points), Severe (25–34 points), and Complete (35–50 points).^19^ We observed Mild, Moderate, Severe, and Complete disabilities in 764 (28.6%), 58 (2.2%), 2 (0.1%), and 0 (0.0%), respectively; therefore, the participants were assigned to two groups: either those without (NDI < 5) or with neck pain (NDI ≥ 5). The reliability and validity of the Japanese version of the NDI were described in a previous study.^20^

### 2.3 Assessment of PIU

Problematic internet use was measured by the 14‐item Compulsive Internet Use Scale (CIUS), which is one of the most frequently internationally adapted psychometric instruments developed to measure compulsive Internet use behavior of private Internet use by using both intra‐ and inter‐assay.^21^ Its 14 items assess the frequency of events (“How often…?”), with a total score ranging from 0 to 56 measured on a five‐point Likert scale (“never”, “seldom”, “sometimes”, “often”, and “very often”). CIUS does not have a predetermined cut off score for PIU.^21^ Considering the relation of higher CIUS score to PIU risk,^22^, ^23^ we sought to examine the graded association of CIUS score and neck pain. Therefore, the scores were divided into quartiles as follows: Quartile 1 = 0–2 points, Quartile 2 = 3–7 points, Quartile 3 = 8–14 points, Quartile 4 = 15–56 points (median: 7, IQR: 2–14). The reliability and validity of the Japanese version of CIUS were described in a previous study.^21^

### 2.4 Assessment of internet usage

Internet usage was measured by asking how many hours on average respondents spent daily using the Internet for private use on weekdays and weekends over the last 90 days.^3^, ^16^ The choices were (1) not at all, (2) daily 1–29 min, (3) daily 30–59 min, (4) daily 60–119 min, (5) daily 120–179 min, (6) daily 180–239 min, (7) daily 240–299 min, (8) daily 300–359 min, or (9) daily ≥360 min. We observed (1)–(9) in 24 (0.9%), 892 (34.5%), 863 (33.4%), 562 (21.8%), 173 (6.7%), 45 (1.7%), 14 (0.5%), 3 (0.1%), and 6 (0.2%), respectively; therefore, these alternatives were categorized into five groups as follows: (1) not at all, (2) daily 1–29 min, (3) daily 30–59 min, (4) daily 60–119 min, (5) daily ≥120 min. The devices (cell phone/PHS, smartphone, tablet, laptop computer, desktop computer) and purpose of Internet use (work, entertainment, online gaming, communication) were assessed based on a questionnaire.

### 2.5 Relevant covariates

The details for assessment of covariates have been published elsewhere.^3^, ^16^, ^24^ A self‐administered survey was performed to obtain information on demographic, social, biological, and psychological factors. Relevant covariates included sex, age (20–29, 30–39, 40–49, 50–59, ≥60 years), total duration of service (<5, 5–9, 10–19, 20–29, ≥30 years), employment position (executive, teacher, lecturer (full‐time), lecturer (part‐time)), school type (junior or senior high school), having children aged ≤18 years, marital status (married), and insomnia and psychological distress. Insomnia was assessed by The Athens Insomnia Scale (AIS), which contains eight items scored on a four‐point Likert scale. Total AIS scores range from 0 to 24, with a higher score indicating greater insomnia symptom severity. The widely accepted cut off score for the diagnosis of insomnia is 6.^25^ Psychological distress was evaluated by Kessler 6 (K6), which consisted of six items measured on a five‐point scale (0–5).^26^, ^27^ The total score range is from 0 to 24, with a higher score indicating more severe mental disorders or mood and anxiety disorders. The cut off point is 5 for K6; therefore, the participants were allocated into two groups: either having no psychological distress (K6 < 5) or having psychological distress (K6 ≥ 5).^28^

### 2.6 Statistical analysis

Demographics and Internet‐related factors between those without neck pain and with neck pain were compared by using the chi‐square test. Logistic regression analysis was used to calculate odds ratio (OR) and 95% confidence interval (CI) of each independent variable for neck pain. The following variables were used as independent variables in this analysis: CIUS category, Internet usage time on weekdays or weekends. Model 1 was the crude univariate model. Model 2 was adjusted for sex and age. Model 3 was additionally adjusted for position at school, insomnia, and psychological distress. Covariates demonstrating significant bivariate associations (*P* < .05) were included in the multivariable models. The selected factors were the same as those showing *P* < .05 in the bivariate association when we chose factors showing *P* < .2 in the bivariate association as covariates being adjusted for multivariable models according to the previous studies on computer usage and neck pain.^29^, ^30^ These covariates were selected a priori because they were considered as potential confounding factors between PIU and physical disorders based on prior reports.^14^, ^15^, ^31^, ^32^ The six variables (sex, age, total duration of service, position at school, insomnia, and psychological distress) showed significant bivariate associations with neck pain (*P* < .05). The absolute value of the correlation coefficients among these variables is all less than 0.7, except for that between age and total duration of service with 0.84. Thus, total duration of service was not selected as a covariate being adjusted for multivariable models in our study. We determined sex, age, position at school, insomnia, and psychological distress as covariates being adjusted for the multivariable models. We performed the sensitivity analysis using the cut off point in CIUS of 21^22^ or 29^33^ defined by the previous studies. We repeated analysis to assess the association of device or purpose of Internet use with neck pain. The Statistical Package for the Social Sciences (Version 21.0, ; SPSS Japan Inc., ) was used for the analyses. Two‐tailed *P* < .05 were considered statistically significant.

## 3 RESULTS

Characteristics of the participants between those with and without neck pain are shown in Table 1. In our study, the prevalence of neck pain was 31.0% (*n* = 800). Compared to those without neck pain, participants with neck pain had a higher prevalence of women in the middle‐aged groups (40–49 and 50–59 years old) with a longer duration of service (20 years or longer) having employment as a teacher with insomnia and psychological distress. Table 2 shows characteristics of the participants' internet‐related factors between those with and without neck pain. The median (interquartile range) of CIUS scores was 7 (2, 14). Participants with neck pain had a higher CIUS category than those without neck pain; however, there was no significant difference with Internet usage on both weekdays and weekends between those with and without neck pain. There was also no significant difference in devices or purposes of Internet use between those with and without neck pain (Table S2).

**TABLE 1:** Characteristics of study participants with and without neck pain, Shimane and Tottori, Japan, 2018

Demographic characteristics	Neck pain		*P* ^a^
	(‐) *n* (%)	(+) *n* (%)	
All	1782 (100)	800 (100)	
Sex, female	570 (32.0)	349 (43.6)	<.001
Age, years			.002
20–29	261 (14.6)	85 (10.6)	
30–39	303 (17.0)	119 (14.9)	
40–49	495 (27.8)	235 (29.4)	
50–59	593 (33.3)	315 (39.4)	
≥60	130 (7.3)	46 (5.8)	
Total duration of service, years			.003
<5	246 (13.8)	74 (9.3)	
5–9	197 (11.1)	77 (9.6)	
10–19	393 (22.1)	167 (20.9)	
20–29	493 (27.7)	247 (30.9)	
≥30	453 (25.4)	235 (29.4)	
Position at school			.04
Principal, vice‐principal	185 (10.4)	67 (8.4)	
General teacher	1275 (71.5)	613 (76.6)	
Full‐time lecturer	230 (12.9)	80 (10.0)	
Part‐time lecturer	92 (5.2)	40 (5.0)	
School type			.79
Junior high school	727 (40.8)	322 (40.3)	
Senior high school	1055 (59.2)	478 (59.8)	
Having children aged ≤18 years	746 (42.6)	340 (43.3)	.75
Marital status, married	1274 (72.1)	583 (73.0)	.63
Insomnia^b^	305 (18.9)	351 (48.0)	<.001
Psychological distress^c^	249 (14.1)	307 (38.9)	<.001

^a^ *P*‐value was assessed using a chi‐square test.

^b^ Insomnia was defined as a score greater than 6 on the Athens Insomnia Scale.

^c^ Psychological distress was defined as more than 5 scores in Kessler 6.

**TABLE 2:** Characteristics of internet‐related factors among study participants with and without neck pain

Internet‐related factor	Neck pain		*P* ^b^
	(−) *n* (%)	(+) *n* (%)	
All	1782 (100)	800 (100)	
CIUS category (score range)			<.001
Q1 (0–2)	522 (29.3)	180 (22.5)	
Q2 (3–7)	450 (25.3)	176 (22.0)	
Q3 (8–14)	449 (25.2)	218 (27.3)	
Q4 (15–56)	361 (20.3)	226 (28.3)	
Median (IQR)	7 (2, 13)	9 (3, 16)	<.001
Internet usage time			
Weekdays (min/day)			.44
No use	19 (1.1)	5 (0.6)	
<30	615 (34.5)	277 (34.6)	
30–60	605 (34.0)	258 (32.3)	
60–120	373 (20.9)	189 (23.6)	
≥120	170 (9.5)	71 (8.9)	
Weekends (min/day)			.78
No use	32 (1.8)	15 (1.9)	
<30	429 (24.1)	200 (25.0)	
30–60	535 (30.0)	235 (29.4)	
60–120	408 (22.9)	168 (21.0)	
≥120	378 (21.2)	182 (22.8)	

Abbreviation: CIUS, compulsive internet use scale; IQR, interquartile range.

^a^ *P*‐value was assessed using a chi‐square test.

Table 3 shows the relationships between Internet usage time or PIU and the odds of neck pain. After adjustment for sex, age, position at school, insomnia, and psychological distress (Model 3), a higher CIUS score was associated with a higher prevalence of neck pain (OR of 4th vs. 1st quartiles, 1.42; 95% CI, 1.07–1.88; Trend *P* = .005; OR per 1‐score higher, 1.02; 95% CI, 1.01–1.04). In the sensitivity analysis (Table S1), compared to those with a CIUS score of less than 21, those with a 21 or higher had significantly higher odds for neck pain (OR, 1.56; 95% CI, 1.12–2.18) (Model 3). The analysis using the cut off value of 29 also showed similar results although the association did not reach statistical significance (OR, 1.68; 95% CI, 0.85–3.31) (Model 3) due to small number of participants with CIUS score of 29 or higher. After adjustment for age and sex, subjects with a longer duration of Internet use on weekdays had a higher prevalence of neck pain (Model 2) (Trend *P* = .03). This positive association disappeared after further adjustment for position at school, insomnia, and psychological distress (Model 3) (Trend *P* = .17). Even subjects who used the Internet less than 30 min per day had almost twofold higher odds of neck pain, although the association did not reach statistical significance (Model 3). The association of neck pain with Internet usage time on weekends was weaker than that with Internet use on weekdays (all models). Neither the devices nor purpose of the Internet use was associated with neck pain after adjusting for confounding factors (Table S3).

**TABLE 3:** Odds ratios for neck pain by CIUS score and Internet usage time

	Number of participants	Number of case (%)	Model 1 (95% CI)	Model 2 (95% CI)	Model 3 (95% CI)
All	2582	800 (31.0)			
CIUS category (score range)					
Q1 (0–2)	702	180 (25.6)	1.00 (ref)	1.00 (ref)	1.00 (ref)
Q2 (3–7)	626	176 (28.1)	1.13 (0.89–1.45)	1.21 (0.94–1.55)	1.17 (0.89–1.53)
Q3 (8–14)	667	218 (32.7)	1.41 (1.11–1.78)	1.59 (1.25–2.02)	1.45 (1.11–1.89)
Q4 (15–56)	587	226 (38.5)	1.82 (1.43–2.30)	2.18 (1.69–2.80)	1.42 (1.07–1.88)
*P* for trend			<.001	<.001	.005
Per 1‐score higher			1.03 (1.02–1.04)	1.04 (1.03–1.06)	1.02 (1.01–1.04)
*P*			<.001	<.001	.001
Internet usage time					
Weekdays (min/day)					
No use	24	5 (20.8)	1.00 (ref)	1.00 (ref)	1.00 (ref)
<30	892	277 (31.1)	1.71 (0.63–4.63)	1.81 (0.66–4.95)	2.11 (0.72–6.21)
30–60	863	258 (29.9)	1.62 (0.60–4.39)	1.91 (0.70–5.22)	2.05 (0.69–6.04)
60–120	562	189 (33.6)	1.93 (0.71–5.24)	2.43 (0.88–6.70)	2.55 (0.86–7.58)
≥120	241	71 (29.5)	1.59 (0.57–4.42)	2.07 (0.73–5.86)	2.30 (0.75–7.05)
*P* for trend			.61	.03	.17
Weekends (min/day)					
No use	47	15 (31.9)	1.00 (ref)	1.00 (ref)	1.00 (ref)
<30	629	200 (31.8)	0.99 (0.53–1.88)	1.07 (0.56–2.04)	0.79 (0.45–1.82)
30–60	770	235 (30.5)	0.94 (0.50–1.76)	1.09 (0.57–2.07)	0.89 (0.45–1.78)
60–120	576	168 (29.2)	0.88 (0.46–1.66)	1.07 (0.56–2.06)	0.86 (0.43–1.74)
≥120	560	182 (32.5)	1.03 (0.54–1.94)	1.36 (0.70–2.63)	1.06 (0.52–2.16)
*P* for trend			.98	.11	.47

Note

All values are expressed as ORs and 95% CIs based on logistic regression. Model 1 was a crude model. Model 2 was adjusted for sex and age. Model 3 was additionally adjusted for position at school, insomnia, and psychological distress.

Abbreviations: CI, confidence interval; CIUS, compulsive internet use scale; OR, odds ratio.

## 4 DISCUSSION

Our study showed that a higher CIUS score was associated with a higher prevalence of neck pain among teachers aged 20 years or older in Japan after adjustment for confounding factors including insomnia and psychological distress. We also showed that a longer time of daily Internet usage on weekdays was positively associated with neck pain among Japanese teachers although the associations did not reach the customary level for designation of statistical significance. The association of neck pain with Internet usage time on weekdays was stronger than that on weekends.

The observed prevalence of neck pain assessed by NDI in our study was similar to that reported among Pakistan teachers (35.6% in 168 women and 110 men; mean age, 32.0 years),^34^ but lower than those from other studies among Polish teachers (43.0% and 47.4% in 840 women and 158 men, respectively; mean age, 38.5 years),^35^ among Polish university students at the medical school (57.3% in 73 women and 39 men; mean age, 22.9 years),^36^ and among Indian university students at the department of computer science (65.8% in 112 women and 388 men aged 18–25 years).^37^ Moreover, in a 1‐year prospective cohort study among Thai, healthy office workers (429 women and 106 men; mean age, 39.2 years), the incident neck pain with disability (NDI ≥ 5) during the follow‐up was 23% (95% CI, 0.15–0.31) with the mean NDI score of 8.4,^38^ and in another 1‐year prospective cohort study among Thai, healthy office workers (501 women and 168 men; mean age, 35.7 years), the incident neck pain during follow‐up period was 21.3% with the mean NDI score of 6.7.^39^ The discrepancy in the prevalent neck pain by NDI may be because of the differences in participants' characteristics (e.g., age, sex, race, occupation) between studies.

In most previous studies, PIU is suggested to be related to psychological problems among schoolteachers. A cross‐sectional study among 1696 junior high school teachers in Japan indicated that those who were at risk of PIU had a positive relationship with one factor of burnout syndrome: depersonalization.^3^ Another cross‐sectional study among 2663 junior and senior high school teachers in Japan proved that teachers with more severe psychological distress, regardless of gender, used the Internet in a more problematic way.^24^ However, to our knowledge, there were few studies on the relationship between PIU and physical disorders, and therefore, this is the first study looking at schoolteachers to examine this issue.

Our results proved that a higher CIUS score was associated with a higher prevalence of neck pain among teachers aged 20 years or older in Japan. Previous studies among mainly adolescents or college students have reported the association of PIU and musculoskeletal pain. A study among 4211 college students aged 16–24 in China found that severe PIU, assessed by the internet addiction test (IAT), was related to musculoskeletal pain in multiple body sites.^14^ However, as the IAT is heavily skewed toward younger people, the CIUS is considered to be a better scale than the IAT for measuring problematic compulsive Internet behavior.^21^ Computer usage time exceeding 2 h/day was also positively associated with neck‐shoulder pain in 6003 Finnish adolescents aged 14–16 years old.^15^ In addition, smartphone addiction rated by the Smartphone Addiction Scale was associated with neck problems and disability among 78 students with a mean age (SD) of 21.3 (1.7) years.^40^ Our findings among Japanese teachers aged 20 years or older, in addition to the abovementioned prior evidence mainly among adolescent or college students, indicate that PIU or longer usage of the Internet is associated with physical disorders such as musculoskeletal pain from adolescence to adulthood.

The exact mechanism of the association between PIU and musculoskeletal pain remains unknown, but most studies have postulated that Internet usage time would mediate this relationship.^13^, ^14^ A link between PIU and excessive Internet usage time was most commonly accepted,^3^, ^41^ indicating the possible mechanism of the association of PIU with development of musculoskeletal pain.^14^ During Internet or smartphone use, such as chatting with a friend by text message or playing games online, users often remain in a fixed position while gripping their smartphones for an extended period of time with wrists extended and pronated, elbows flexed, and head down.^14^ These poor postural habits could lead to strain on the muscles, tendons, and disks, consequently resulting in neck, shoulder, elbow, and wrist/hand pain.^14^ In our study, compared with non‐Internet users, almost twice higher odds of neck pain were found among weekday online users and were less apparent on weekends. This might be because the Internet is used in a more relaxed state on weekends, which leads to reducing the use of neck muscles.^42^ Musculoskeletal pain has been reported to be associated with not only the length of Internet use, but also poor posture,^43^, ^44^ lightning,^45^ mental status,^31^, ^46^ and other causes,^43^ suggesting the complex factors in the development of neck pain. The exact explanation linking neck pain and PIU remains unclear and warrants further research.

The strengths of our study include the point that, to our knowledge, this study represents the first report on the association between PIU or Internet usage time and neck pain, one of the most prevalent musculoskeletal symptoms,^17^, ^18^ among adults aged 20 years or older. In addition, our measurements for main variables used standardized methods with eligible validation for evaluating both PIU and neck pain.

However, the limitations of our study warrant consideration. First, a causative link between PIU and neck pain could not be established because of a cross‐sectional design. Second, the possibility of potential selection bias needs to be considered when especially mentioning the prevalence of PIU, long Internet usage time, and neck pain due to low response rate (33.2%) for the questionnaire. Third, the number of people who do not use the Internet (reference) on weekdays or weekends was very small, thus yielding relatively low statistical power to detect true associations. The association between the amount of Internet usage especially on weekdays and neck pain did not reach the customary level for designation of statistical significance with a wide 95% CI, possibly as a result of the small sample size. Fourth, we carefully controlled for major known confounders; however, our findings may be partly explained by differences in unknown confounders. Finally, as only schoolteachers aged 20 years or older in Japanese rural areas were included in the analysis, our results cannot be generalized to other populations.

## 5 CONCLUSION

Problematic internet use based on a high score on the CIUS was positively associated with neck pain among Japanese junior and senior high school teachers aged 20 years and older, even after adjusting for insomnia and psychological distress. We showed that PIU was one of the independent factors for neck pain among adults. Our results suggest that adults with PIU may be at risk of physical disorders *in addition to mental* health disorders. In future research, it will be necessary to explore the causative links between PIU and musculoskeletal pain with prospective cohort or other interventional studies.

## DISCLOSURE

*Approval of the research protocol*: This survey was approved by the Institutional Review Board of Shimane University (No. 3274, Approved on July 24, 2018) and Okayama University (No.2020–027, Approved on January 31, 2020). *Informed consent*: Informed consent was waived because of the anonymous and voluntary nature of the present study. *Registry and the registration no*. *of the study*/*trial*: N/A. *Animal studies*: N/A. *Conflict of interest*: None.

## AUTHOR CONTRIBUTIONS

Takashi Hisamatsu, Mari Fukuda, Hideki Tsumura, Rina Tsuchie, Masako Suzuki, Nagisa Sugaya, Koshi Nakamura, Kenzo Takahashi, and Hideyuki Kanda contributed to the conception and design. Mari Fukuda, Hideki Tsumura, Rina Tsuchie, Masako Suzuki, Nagisa Sugaya, Koshi Nakamura, Kenzo Takahashi, and Hideyuki Kanda collected the data. Rina Tanabe, Takashi Hisamatsu, Mari Fukuda, and Hideyuki Kanda analyzed and interpreted the data. Rina Tanabe drafted the article. All authors critically revised the article for intellectual content. All authors approved the final version.

## Supporting information

Table S1‐3

## Data Availability

The data that support the findings of this study are available from the corresponding author upon reasonable request.
