# Supplementary material for: The association between problematic internet use and neck pain among Japanese schoolteachers
Source: J Occup Health. 2021 Dec 9;63(1):e12298. doi: 10.1002/1348-9585.12298 (PMC8661443; doi:10.1002/1348-9585.12298)
Supplement: Supplementary file 1 — Table S1‐3 [file JOH2-63-e12298-s001.docx]

**Supplementary table 1.** Odds ratios for neck pain by cut off score of 21 or 29 in CIUS

|  | Number of participants | Number of case (%) | Model 1 (95%CI) | Model 2 (95%CI) | Model 3 (95%CI) |
| --- | --- | --- | --- | --- | --- |
| All | 2582 | 800 (31.0) |  |  |  |
| CIUS category |  |  |  |  |  |
| None (< 21) | 2346 | 699 (29.6) | 1.00 (ref) | 1.00 (ref) | 1.00 (ref) |
| Problematic user (≥21) | 218 | 101 (46.3) | 2.06 (1.55-2.72) | 2.30 (1.72-3.07) | 1.56 (1.12-2.18) |
| CIUS category |  |  |  |  |  |
| None (< 29) | 2539 | 778 (30.6) | 1.00 (ref) | 1.00 (ref) | 1.00 (ref) |
| Problematic user (≥29) | 43 | 22 (51.2) | 2.37 (1.30-4.34) | 2.84 (1.53-5.26) | 1.68 (0.85-3.31) |

All values are expressed as ORs and 95% CIs based on logistic regression. Model 1 was a crude model. Model 2 was adjusted for sex and age. Model 3 was additionally adjusted for position at school, insomnia, and psychological distress.

Abbreviation: Confidence Interval (CI); Compulsive Internet Use Scale (CIUS); Odds ratio (OR).

**Supplementary Table 2.** Characteristics of devices and purposes of Internet use among study participants with and without neck pain

|  | Neck pain | |  |
| --- | --- | --- | --- |
| Device and purpose | (－) n (%) | (＋) n (%) | P^†^ |
| All | 1782 (100) | 800 (100) |  |
| Device |  |  |  |
| Cell phone/PHS | 140 (7.9) | 71 (8.9) | 0.38 |
| Smartphone | 1485 (83.3) | 646 (80.8) | 0.11 |
| Tablet | 729 (40.9) | 338 (42.3) | 0.52 |
| Laptop computer | 1454 (81.6) | 664 (83.0) | 0.39 |
| Desktop computer | 664 (37.3) | 304 (38.0) | 0.72 |
| Number of devices |  |  | 0.69 |
| None or 1 device | 171 (9.6) | 81 (10.1) |  |
| 2 devices | 765 (42.9) | 336 (42.0) |  |
| 3 devices | 619 (34.7) | 266 (33.3) |  |
| 4 devices | 215 (12.1) | 112 (14.0) |  |
| 5 devices | 12 (0.7) | 5 (0.6) |  |
| Purpose |  |  |  |
| Work | 1685 (94.6) | 768 (96.0) | 0.12 |
| Entertainment | 1669 (93.7) | 739 (92.4) | 0.23 |
| Online gaming | 287 (16.1) | 126 (15.8) | 0.82 |
| Communication | 1336 (75.0) | 615 (76.9) | 0.30 |

^†^ P-value was assessed using a chi-square test.

Abbreviation: Personal handy-phone system (PHS).

**Supplementary Table 3.** Odds ratios for neck pain by the devices and purposes of Internet use

|  | Number of participants | Number of case (%) | Model 1 (95%CI) | Model 2 (95%CI) | Model 3 (95%CI) |
| --- | --- | --- | --- | --- | --- |
| All | 2582 | 800 (31.0) |  |  |  |
| Device |  |  |  |  |  |
| Cell phone/PHS |  |  |  |  |  |
| － | 2371 | 729 (30.5) | 1.00 (ref) | 1.00 (ref) | 1.00 (ref) |
| ＋ | 211 | 71 (31.7) | 1.14 (0.85-1.54) | 1.12 (0.83-1.51) | 1.04 (0.74-1.46) |
| P |  |  | 0.38 | 0.47 | 0.83 |
| Smartphone |  |  |  |  |  |
| － | 451 | 154 (34.1) | 1.00 (ref) | 1.00 (ref) | 1.00 (ref) |
| ＋ | 2131 | 646 (30.3) | 0.84 (0.68-1.04) | 0.87 (0.69-1.09) | 0.88 (0.68-1.13) |
| P |  |  | 0.11 | 0.21 | 0.31 |
| Tablet |  |  |  |  |  |
| － | 1515 | 462 (30.5) | 1.00 (ref) | 1.00 (ref) | 1.00 (ref) |
| ＋ | 1067 | 338 (31.7) | 1.06 (0.89-1.25) | 1.12 (0.95-1.33) | 1.11 (0.92-1.35) |
| P |  |  | 0.52 | 0.19 | 0.29 |
| Laptop computer |  |  |  |  |  |
| － | 464 | 136 (29.3) | 1.00 (ref) | 1.00 (ref) | 1.00 (ref) |
| ＋ | 2118 | 664 (31.4) | 1.10 (0.88-1.37) | 1.14 (0.91-1.42) | 1.15 (0.90-1.48) |
| P |  |  | 0.39 | 0.26 | 0.26 |
| Desktop computer |  |  |  |  |  |
| － | 1614 | 496 (30.7) | 1.00 (ref) | 1.00 (ref) | 1.00 (ref) |
| ＋ | 968 | 304 (31.4) | 1.03 (0.87-1.23) | 1.05 (0.88-1.25) | 1.00 (0.82-1.21) |
| P |  |  | 0.72 | 0.58 | 0.96 |
| The number of devices |  |  |  |  |  |
| none or a device | 252 | 81 (32.1) | 1.00 (ref) | 1.00 (ref) | 1.00 (ref) |
| 2 devices | 1101 | 336 (30.5) | 0.93 (0.69-1.24) | 0.94 (0.69-1.26) | 0.90 (0.64-1.26) |
| 3 devices | 885 | 266 (30.1) | 0.91 (0.67-1.23) | 0.96 (0.71-1.30) | 0.93 (0.66-1.31) |
| 4 devices | 327 | 112 (34.3) | 1.10 (0.78-1.56) | 1.22 (0.86-1.74) | 1.12 (0.75-1.67) |
| 5 devices | 17 | 5 (29.4) | 0.88 (0.30-2.58) | 1.00 (0.34-2.96) | 0.79 (0.22-2.80) |
| P |  |  | 0.64 | 0.22 | 0.48 |
| Purpose |  |  |  |  |  |
| Work |  |  |  |  |  |
| － | 129 | 32 (24.8) | 1.00 (ref) | 1.00 (ref) | 1.00 (ref) |
| ＋ | 2453 | 768 (31.3) | 1.38 (0.92-2.08) | 1.47 (0.97-2.22) | 1.35 (0.85-2.15) |
| P |  |  | 0.12 | 0.68 | 0.20 |
| Entertainment |  |  |  |  |  |
| － | 174 | 61 (35.1) | 1.00 (ref) | 1.00 (ref) | 1.00 (ref) |
| ＋ | 2408 | 739 (30.7) | 0.82 (0.59-1.13) | 0.84 (0.60-1.16) | 0.84 (0.58-1.21) |
| P |  |  | 0.23 | 0.28 | 0.35 |
| Online game |  |  |  |  |  |
| － | 2169 | 674 (31.1) | 1.00 (ref) | 1.00 (ref) | 1.00 (ref) |
| ＋ | 413 | 126 (30.5) | 1.11 (0.91-1.35) | 1.12 (0.91-1.37) | 1.08 (0.83-1.41) |
| P |  |  | 0.30 | 0.30 | 0.57 |
| Communication |  |  |  |  |  |
| － | 631 | 185 (29.3) | 1.00 (ref) | 1.00 (ref) | 1.00 (ref) |
| ＋ | 1951 | 615 (31.5) | 0.97 (0.78-1.22) | 1.09 (0.86-1.39) | 1.08 (0.86-1.36) |
| P |  |  | 0.82 | 0.47 | 0.49 |

All values are expressed as ORs and 95% CIs based on logistic regression. Model 1 was a crude model. Model 2 was adjusted for sex and age. Model 3 was additionally adjusted for position at school, insomnia, and psychological distress.

Abbreviation: Confidence interval (CI); Odds ratio (OR); Personal handy-phone system (PHS).
